# *Ascaris suum* excretory/secretory products differentially modulate porcine dendritic cell subsets

**DOI:** 10.3389/fimmu.2022.1012717

**Published:** 2022-11-10

**Authors:** Benjamin Hamid, Friederike Ebner, Lalita Bechtold, Arkadi Kundik, Sebastian Rausch, Susanne Hartmann

**Affiliations:** Institute of Immunology, Department of Veterinary Medicine, Freie Universität Berlin, Berlin, Germany

**Keywords:** *Ascaris suum*, blood, dendritic cells, excretory/secretory products, helminth, immunomodulation, pigs

## Abstract

Helminths produce excretory/secretory products (E/S) which can modulate the immune responses of their hosts. Dendritic cells (DC) are essential for initiating the host T cell response and are thus potential targets for modulation by helminth E/S. Here we study immunomodulation of porcine peripheral blood DC subsets following *ex vivo* stimulation with E/S from *Ascaris suum*, a common helminth of pigs with considerable public health and economic importance. Our data showed that the relative frequencies of DC subsets in porcine blood differ, with plasmacytoid DC (pDC) being the most prominent in healthy 6-month-old pigs. pDC are an important cytokine source, and we found that *A. suum* E/S suppressed production of the type 1 cytokines IL-12p40 and TNF-α by this subset following toll-like receptor (TLR) ligation. In contrast, conventional DC (cDC) are more efficient antigen presenters, and the expression of CD80/86, costimulatory molecules essential for efficient antigen presentation, were modulated differentially by *A. suum* E/S between cDC subsets. CD80/86 expression by type 1 cDC (cDC1) following TLR ligation was greatly suppressed by the addition of *A. suum* E/S, while CD80/86 expression by type 2 cDC (cDC2) was upregulated by *A. suum* E/S. Further, we found that IFN-γ production by natural killer (NK) cells following IL-12 and IL-18 stimulation was suppressed by *A. suum* E/S. Finally, in the presence of E/S, IFN-γ production by CD4+ T cells co-cultured with autologous blood-derived DC was significantly impaired. Together, these data provide a coherent picture regarding the regulation of type 1 responses by *A. suum* E/S. Responsiveness of pDC and cDC1 to microbial ligands is reduced in the presence of E/S, effector functions of Th1 cells are impaired, and cytokine-driven IFN-γ release by NK cells is limited.

## Introduction

Helminths are complex in their biology and life cycles, with different species causing different pathology during infection. Yet, most helminths induce a common immune response, characterized by the polarization of naïve CD4+ T cells towards type 2 T helper cell (Th2) and regulatory T cell phenotypes during chronic infection. Chronicity of infection is often associated with hyporesponsiveness towards helminth antigens, which is mutually beneficial to host and helminth, by preventing tissue damage from excessive inflammation while inhibiting elimination of the parasite. This immunomodulation can result in adverse secondary consequences, such as poor control of co-infections by intracellular pathogens, but can also be beneficial in dampening allergic and auto-immune diseases (1–3).

*Ascaris suum* is a common helminth of pigs which causes considerable economic losses in livestock, but also impacts public health due to its zoonotic potential (4–6). Moreover, *A. suum* is closely related to *A. lumbricoides*, which is the most common cause of helminth infection in humans worldwide (7). Indeed, in light of extensive parasitological and molecular similarities, some authors consider *A. suum* and *A. lumbricoides* as a single species (8). Therefore, better understanding immune modulation by *A. suum* may provide insight on immunomodulation during *Ascaris* infection in both pigs and humans. This knowledge may inform the handling of further immune challenges in both hosts, such as treatment of secondary infections, vaccination strategies, and management of hypersensitivity reactions.

Dendritic cells (DC) are essential for initiating and influencing the direction of the subsequent T cell response to parasite infection and hence constitute an important target for immune modulation by helminths (9). The DC population found *in vivo* consists of a diverse range of cell subsets with different phenotypic profiles. These subpopulations are difficult to define on the basis of functionality due to considerable plasticity and phenotypic differences between DC from different anatomical locations and species. Therefore, DC subsets are defined on the basis of ontogeny, with plasmacytoid DC (pDC) and pre-conventional DC (pre-cDC) differentiating from common DC progenitors, and pre-cDC subsequently dividing into type 1 (cDC1) and type 2 (cDC2) lineages (10). cDC1, cDC2 and pDC are collectively termed *bona fide* DC. cDC from human blood are highly efficient antigen presenters, with cDC1 and cDC2 being equally potent at presentation to CD4+ and CD8+ T cells. pDC can also present antigens, but are highly specialized for responding to viral infection with IFN-α production (11).

Prior research targeting the immunomodulatory effects of *Ascaris* and DC primarily relied on the use of murine bone marrow-derived (bmDC) (12, 13) and human monocyte-derived DC (moDC) (14, 15) generated *in vitro*. bmDC exhibit an immature phenotype in comparison to peripheral blood DC (16), whereas moDC differentiate from monocytes *in vivo* at sites of inflammation, but are not present during steady-state conditions (10). Therefore, these *in vitro* generated DC may not be representative of the DC present *in vivo* during *Ascaris* infection. Here we phenotyped, for the first-time to our knowledge, *bona fide* DC subsets from pigs – the natural host of *A. suum* – following *ex vivo* stimulation with *A. suum* excretory/secretory products (E/S). In parallel, we assessed the consequences of combined *A. suum* E/S and toll-like receptor (TLR) ligand exposure on cytokine production and markers of antigen presentation by *bona fide* DC subsets.

DC, as professional antigen presenting cells, are essential for inducing T cell polarization and determining the direction of the subsequent adaptive immune response. However other innate cells, notably natural killer (NK) cells, also contribute to directing downstream immune responses through cytokine production. DC-NK cross-talk is important for amplifying type 1 signals, as NK cells produce IFN-γ upon stimulation with DC-derived IL-12 and IL-18, with this IFN-γ fostering further IL-12 production by DC (17, 18). Hence, in parallel we investigated the capacity of *A. suum* E/S to modulate cytokine-driven IFN-γ production by NK cells. Finally, we aimed to decipher the impact of *A. suum* E/S exposure on the ability of DC to activate T helper cells, as this is the primary task of DC (19).

Our data show that *A. suum* E/S differentially modulate CD80/86 expression by porcine cDC and suppress IL-12p40 and TNF-α production by pDC. We further demonstrate that E/S exposure restrains IFN-γ production by NK cells, independently of DC, and finally, impairs IFN-γ production by CD4+ T cells in co-culture with TLR-ligated DC.

## Materials and methods

### Peripheral blood mononuclear cell isolation

Whole peripheral blood was collected from 6-month old pigs upon exsanguination at the slaughterhouse into bottles prefilled with 0.5 M EDTA for a final concentration of 1.5 mg EDTA per 1 ml blood. Blood was diluted in 0.9% NaCl solution at 1:1 ratio, layered over Pancoll (1.077 g/ml density; PAN-Biotech GmbH; Aidenbach, BY, Germany), and density gradient centrifugation was performed. The PBMC layer was collected, washed in 0.9% NaCl solution, and remaining erythrocytes were destroyed by incubating in lysis solution (H_2_O plus 0.01 M KHCO_3_, 0.155 M NH_4_Cl and 0.1 M EDTA, pH 7.5) at room temperature for 5 minutes. PBMC were washed twice in complete Iscove’s modified Dulbecco’s medium (cIMDM; IMDM with stable glutamine, 25 nM HEPES and 3.024 g/L NaHCO3, plus 10% fetal bovine serum (FBS), 100 U/ml penicillin and 100 μg/ml streptomycin; all: PAN-Biotech GmbH; Aidenbach, BY, Germany) and prepared for experiments as described. Each porcine blood sample was collected on a different day, and experiments were performed independently on each sample, according to the same protocols. The DC subset, NK cell and monocyte stimulations presented in **Figures 2**–**4** and Supplementary **Figures S1**-**3** were performed on each blood sample in parallel. The DC – T cell co-culture experiments presented in **Figure 5** were subsequently performed on different blood samples.

### Peripheral blood DC subset and monocyte isolation

**Figure 1:**
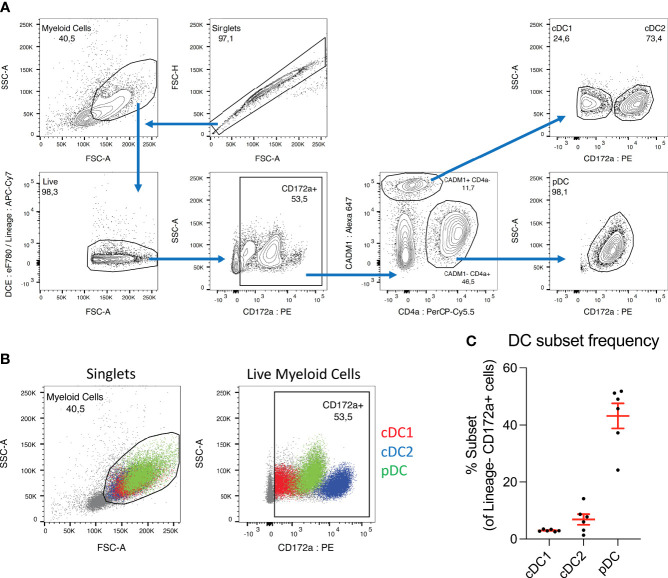
pDC dominate the DC population in porcine blood. **(A)** Sorting strategy used to isolate dendritic cell subsets from porcine PBMC. CD3+ and γδ-TCR1+ T cells, CD21+ and IgM+ B cells and CD14+ monocytes were first depleted by magnetic cell separation, and the negative fraction was then taken forward for FACS sorting. Cell aggregates were excluded and myeloid cells selected based on size (FSC-A) and granularity (SSC-A). Cells were treated with a fixable viability dye, and labelled, dead cells excluded (DCE: eF780). In parallel, any remaining CD3+, γδ-TCR1+, CD21+, IgM+ or CD14+ cells were labelled and excluded (Lineage: APC-Cy7). CD172a+ cells were selected, and within this population, CADM1+ CD4a- cDC were selected and further divided into CD172a low cDC1 and CD172a high cDC2. The CADM1- CD4a+ cell population was also selected, which consist almost entirely of CD172a mid pDC. **(B)** Overlays of sorted DC subsets within myeloid cell (left) and CD172a+ (right) gates. Presented with cDC2 (blue) as the bottom layer, with cDC1 (red) above and pDC (green) on top. Overlays were used for adjusting gating prior to sorting. **(C)** Percentages of each DC subset within the CD172a+ gate. Each dot represents one pig, n = 6.

Porcine *bona fide* DC subsets were sorted from PBMC according to an adapted version of a previously published protocol (20, 21). In brief, CD3ε+, γδ-TCR1+, CD21+ and IgM+ PBMC were first depleted by magnetic cell separation using an autoMACS Pro Separator (Miltenyi Biotec; Bergisch Gladbach NW, Germany; RRID : SCR_018596), followed by a second round of magnetic cell separation to isolate CD14+ monocytes. Cells remaining in the negative fraction were re-labelled with anti-CD3ε, γδ-TCR1, CD21, IgM and CD14 antibodies, and with fixable viability dye (eFluor 780; ThermoFisher; Waltham, MA, USA), as well as with anti-CD172a, CADM1 and CD4a antibodies. DC subsets were identified and sorted from within the live, CD3ε-, γδ-TCR1-, CD21-, IgM- CD14- population by florescence-activated cell sorting (FACS) using the FACSAria III instrument (BD Biosciences; Franklin Likes, NJ, USA; RRID: SCR_016695). cDC1 were defined as CADM1+ CD4a- CD172a^low^, cDC2 were defined as CADM1+ CD4a- CD172a^high^, and pDC were defined as CADM1- CD4a+ CD172a^mid^. Sorting strategy is displayed in **Figure 1A**, antibody details are available in **Supplementary Table S1**.

### *Ascaris suum* E/S collection

*A. suum* worms were collected from the intestines of 6-month old pigs at the slaughterhouse, immediately after euthanasia. Motile, female worms were separated and washed 5 times in 0.9% NaCl solution, for at least 30 seconds per wash with gentle agitation. These were then cultured at a density of 1 worm per 100 ml for 24h at 37°C in culture solution consisting of Hank’s balanced salt solution (HBSS) without glucose, supplemented with 200 U/ml penicillin, 200 μg/ml streptomycin, 2.5 μg/ml amphotericin and 50 μg/ml gentamycin (all antibiotics: PAN-Biotech GmbH; Aidenbach, BY, Germany). Worms were then transferred to fresh culture solution and incubated for a further 24h. Worms were discarded and culture solution from the second 24h was left at 4°C overnight for sediment to settle. The supernatant was removed and centrifuged at 10,000 x g to pellet further particulate matter, and supernatant was sterilized using a 450 nm filter. Sterile flow-through was concentrated using Vivaspin columns (5kDa molecular weight cut-off; Sartorius; Göttingen, Germany), washed with PBS (PAN-Biotech GmbH; Aidenbach, BY, Germany) and passed through 200 nm filters. Protein concentration was measured by bicinchoninic acid test (ThermoFisher; Waltham, MA, USA) and E/S diluted in PBS to a stock concentration of 1 mg/ml. Endotoxin content was determined using the limulus amoebocyte lysate assay (Lonza; Basel, Switzerland) and measured at 10045 EU/ml stock. Pooled E/S from 60 worms was used for the experiments.

### *In vitro* stimulation of DC and monocytes

Peripheral blood DC subsets, counted during sorting, were each seeded at a density of 1x10^6^ cells/ml. In parallel, magnetically separated CD14+ monocytes were counted using a CASY automated cell counter (Roche; Basel, Switzerland; RRID : SCR_002080) and also seeded at a density of 1x10^6^ cells/ml. Cell culture media consisted of either cIMDM alone, or cIMDM supplemented with 100 μg/ml *A. suum* E/S and/or 100 ng/ml lipopolysaccharide (LPS; Sigma-Aldrich; St. Louis, MO, USA), 500 ng/ml poly(I:C), and 300 ng/ml R848 (both: *In vivo*gen; San Diego, CA, USA). Poly(I:C), LPS and R848 were administered in combination to simultaneously stimulate TLR 3, 4, 7 and 8. All cells were incubated for 24 hours at 37°C, after which cells were pelleted by centrifugation, and pDC and monocyte supernatants were frozen at -20°C for ELISA. cDC1, cDC2, pDC and monocytes were then all analyzed by flow cytometry.

### Assessment of IFN-γ production by NK cells

Unsorted PBMC were counted using a CASY automated cell counter, seeded at a density of 1x10^6^ cells/ml and incubated for 12 hours at 37°C. Cell culture media consisted of either cIMDM alone, or cIMDM supplemented with 100 μg/ml *A. suum* E/S and/or 100 ng/ml porcine IL-12p70 and 100 ng/ml porcine IL-18 (both: R&D Systems; Minneapolis, MN, USA). All cultures were additionally treated with brefeldin A (3 μg/ml; ThermoFisher; Waltham, MA, USA) immediately following stimulation. After incubation NK cells were analyzed for IFN-γ production by flow cytometry.

### DC – T cell co-culture

All DC subsets isolated from a single pig, counted during sorting, were combined and seeded at a density of 1x10^6^ cells/ml in cIMDM. DC were left untreated or treated with 100 μg/ml *A. suum* E/S and/or 100 ng/ml LPS, 500 ng/ml poly(I:C) and 300 ng/ml R848, and incubated at 37°C. After 18 hours, an equal volume of unsorted autologous PBMC was added to each DC culture, at a density of 1x10^6^ cells/ml. PBMC cell density was measured by CASY automated cell counter. All cultures were treated with 2 μg/ml staphylococcal enterotoxin B (SEB; Sigma-Aldrich; St. Louis, MO, USA) to cross-link MHC-II with T cell receptor Vβ chains, and B7 costimulatory molecules with CD28, and incubated for a further 18 hours. Brefeldin A (3 μg/ml) was added for the final 12 hours. This was followed by surface and intracellular labelling and analysis by flow cytometry.

### FACS analysis of stimulated cells

After stimulation, cDC1, cDC2, pDC and monocytes were labelled with CD152muIg - a fusion protein consisting of cross-reactive human CD152, which binds to porcine CD80 and CD86 (22, 23), and a murine IgG2a-isotype constant region. CD152muIg was subsequently labelled with anti-mouse IgG2a-Brilliant Violet 605. Following this, cells were also labelled with anti-pig CD172a-PE, SLA-DR-FITC, and fixable viability dye in eFluor 780. Cells were fixed using IC fixation buffer (ThermoFisher; Waltham, MA, USA), and stored at 4°C overnight prior to flow cytometry analysis.

To analyze NK cells, stimulated PBMC were labelled with murine IgG1-isotype anti-pig CD172a and IgG2a-isotype anti-pig CD8a, followed by anti-mouse IgG1-APC-Cy7 and IgG2a-BV605. Cells were then labelled with anti-pig CD3-PerCP-Cy5.5, CD16-FITC and fixable viability dye in eFluor 506 (ThermoFisher; Waltham, MA, USA), fixed with IC fixation buffer, and stored at 4°C overnight. Cells were intracellularly labelled with anti-pig IFN-γ-PE immediately prior to analysis. To inform appropriate gating on IFN-γ+ cells, an FMO control was prepared using IL-12p70 and IL-18-stimulated PBMC, which were labelled as described but with the omission of anti-pig IFN-γ-PE.

DC – T cell co-cultures were labelled with murine IgG1-isotype anti-pig CD172a, followed by anti-mouse IgG1-APC-Cy7. They were then labelled with anti-pig CD3e-PerCP-Cy5.5, CD4a-Alexa 647, and fixable viability dye in eFluor 780. Cells were fixed in IC fixation buffer, and then labelled intracellularly with anti-pig IFN-γ-PE and IL-4-Brilliant Violet 421 immediately before flow cytometry analysis. All flow cytometry was performed using the FACSAria III instrument and FlowJo analysis software (FlowJo, LLC; Ashland, OR, USA; RRID: SCR_008520). All antibody details are available in **Supplementary Table S1**.

### Cytokine quantification by ELISA

Porcine IL-12p40, TNF-α, IL-4 and IL-10 concentrations were analyzed in the supernatants of cultured pDC and monocytes using commercially available ELISA kits (all: R&D Systems; Minneapolis, MN, USA; catalog numbers: DY912, DY690B, DY654, DY693) according to manufacturer’s instructions. pDC supernatants were diluted 1:2 in cIMDM prior to measurement of IL-4 (lower limit of detection (LLD): 0.312 ng/ml) and IL-10 (LLD: 0.0468 ng/ml), 1:10 for analysis of IL-12p40 (LLD: 0.781 ng/ml) concentrations and 1:20 prior to TNF-α (LLD: 0.626 ng/ml) quantification. Monocyte supernatants were diluted 1:4 prior to quantification of TNF-α concentrations (LLD: 0.1252 ng/ml), and not diluted before analysis of IL-4 (LLD: 0.156 ng/ml), IL-10 (LLD: 0.0234 ng/ml) or IL-12p40 (0.0781 ng/ml).

To test for direct degradation of IL-12 by *Ascaris* E/S, 0, 50, 100 and 200 ng/ml recombinant porcine IL-12p70 was incubated at 37°C for 24h. Diluent consisted of cIMDM alone, or cIMDM supplemented with 50, 100 or 200 μg/ml E/S. Post-incubation, IL-12p40 concentrations were analyzed by ELISA. All samples were diluted 1:20 in cIMDM prior to measurement (LLD: 1.562 ng/ml).

### Statistical data analysis

Statistical analysis was performed using GraphPad Prism 9 software (RRID: SCR_002798). All datasets were analyzed for Gaussian distribution by Shapiro-Wilk test, and for all experiments the majority of treatment groups passed the normality assessment (p ≥ 0.05; **Supplementary Table S2**). Subsequently differences between treatment groups were analyzed by paired t test, with Bonferroni correction to account for multiple testing.

## Results

### pDC dominate the DC population in porcine blood

To investigate the phenotype of porcine *bona fide* DC stimulated by *A. suum* E/S, we isolated peripheral blood DC subsets from 6-month old pigs. PBMC depleted of monocytes, T- and B cells were further sorted by FACS according to the gating strategy indicated in **Figure 1A**. Within the CD3e- γδ-TCR1- CD21- IgM- CD14- “DC-rich” population, CADM1+ CD4a- were defined as cDC and subdivided into CD172a^low^ cDC1 and, CD172a^high^ cDC2. pDC were defined as CADM1- CD4a+ CD172a^mid^.

Previous work reported cDC2 as the most prominent DC subset in adult human PBMC during steady-state conditions, followed by pDC at a 10 times lower frequency, and cDC1 slightly less numerous than pDC (24). Although direct comparison is difficult due to the use of different cell markers, in PBMC from healthy 6-month-old pigs we observe strikingly different proportions. Within the porcine CD3e- γδ-TCR1- CD21- IgM- CD14- CD172a+ population (**Figure 1B**, second panel), cDC1 accounted for a mean of 3.0 ± 0.37%, compared to 6.8 ± 4.6% for cDC2 and 43 ± 10% for pDC (**Figure 1C**). Hence, while the cDC2 population remains larger than the cDC1 population in porcine blood, the difference is relatively small in comparison to humans. Interestingly, we found pDC to be by far the largest DC sub-population in porcine PBMC, approximately 14 times more frequent than cDC1. The CD3e- γδ-TCR1- CD21- IgM- CD14- CD172a+ population is not a pure DC population and hence DC subsets frequencies do not add up to 100%. Of note, it was recently demonstrated that unlike other species, the majority of porcine pDC express NKp46 (25). Hence, the depletion of NKp46+ cells from the DC-rich fraction applied in our earlier studies was omitted from the current sorting protocol. Consequently, a substantially higher yield of pDC was obtained compared to our previous work (20). NK cells were still excluded by gating on CD172a+ cells.

### *Ascaris* E/S treatment selectively reduces pDC viability, and inhibits enhanced cDC1 longevity following TLR ligation

**Figure 2:**
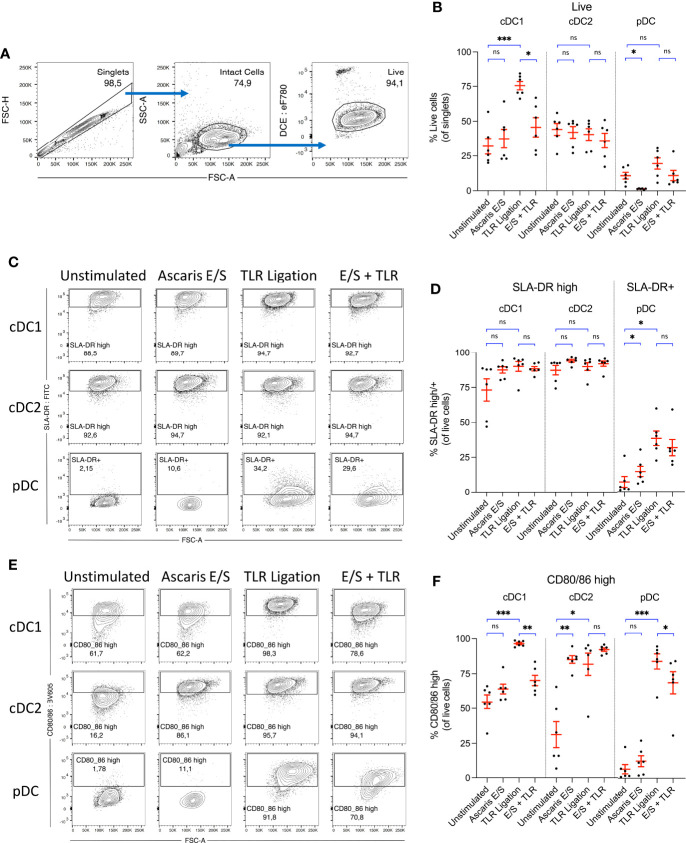
*Ascaris* E/S strongly enhances CD80/86 expression by porcine cDC2, but powerfully inhibits upregulation of CD80/86 by cDC1 following TLR ligation. **(A)** Representative gating strategy used to identify live, intact cells following 24h incubation of sorted DC subsets. Cell aggregates were excluded, and then intact DC were selected based on size (FSC-A) and granularity (SSC-A). Cells were stained with a fixable viability dye, and dead cells were excluded. **(B)** Percentages of viable cells within the total singlet population for each DC subset following incubation without stimulation, stimulation with *Ascaris* E/S, TLR ligation (100 ng/ml LPS, 500 ng/ml poly(I:C) and 300 ng/ml R848), or combined E/S and TLR stimulation. **(C)** Representative gating on SLA-DR high cDC1 and cDC2, and SLA-DR+ pDC following each treatment outlined above. **(D)** Percentages of SLA-DR high cDC1 and cDC2, and SLA-DR+ pDC following each treatment. **(E)** Representative gating on CD80/86 high cells within each DC subset, following each treatment outlined above. **(F)** Percentages of CD80/86 high cells for each DC subset and treatment. **(B, D, F)** Each dot represents a single pig, n = 6. Asterisks indicate statistical significance by paired t test with Bonferroni correction; *p ≤ 0.017, **p ≤ 0.0033, ***p ≤ 0.00033. ns, not significant.

Sorted DC were stimulated for 24 hours with E/S alone, or the TLR ligands LPS, poly(I:C) and R848, or both E/S and TLR ligands combined, and then analyzed by flow cytometry. After exclusion of cell aggregates, intact cells were identified based on size and granularity, followed by dead cell exclusion using a cell viability dye (**Figure 2A**). Differences in cell viability between the DC subsets and treatment groups were readily apparent (**Figure 2B**). 32 ± 14% of unstimulated cDC1 survived after 24h incubation, compared to 44 ± 10% of unstimulated cDC2 and 11 ± 6.0% of unstimulated pDC. These figures are broadly in line with those previously published for murine splenic DC, where 15% of (CD8+) cDC1 survived following 18h incubation, compared to 70% of (CD8-) cDC2 and 25% of pDC (26).

Interestingly, following stimulation with TLR ligands, survival of cDC1 was greatly enhanced, with 75 ± 7.4% of cells viable after 24h incubation. In contrast, there was no enhanced survival of cDC2 following TLR ligation, and only a small, nonsignificant enhancement of pDC survival. Following treatment with *Ascaris* E/S, cDC1 and cDC2 viability were both unaffected. Strikingly though, treatment of pDC with *A. suum* E/S alone consistently induced cell death, with viability significantly reduced to 1.4 ± 0.46% after 24h. Further, addition of E/S to TLR stimulated cDC1 significantly inhibited the TLR-driven enhancement of cDC1 survival. pDC treated with TLR ligation and *Ascaris* E/S also showed a reversal of the small increase in cell survival observed following TLR ligation alone. Therefore, TLR activation greatly enhances cDC1 survival, while treatment with *A. suum* E/S is cytotoxic to pDC and inhibits enhanced survival of TLR-ligated cDC1.

### cDC constitutively express high levels of SLA-DR, while SLA-DR expression is only modestly upregulated by pDC following *Ascaris* E/S stimulation

Next, we analyzed surface expression of SLA-DR. In line with earlier reports (21, 27) both cDC subsets displayed high constitutive SLA-DR expression (cDC1 = 73 ± 19%; cDC2 = 87 ± 8.2%), whereas only few unstimulated pDC (7.1 ± 9.5%) expressed SLA-DR at a low level (**Figures 2C, D**). However, upon stimulation with *A. suum* E/S or TLR ligands, pDC displayed a significant upregulation of SLA-DR expression. Levels of SLA-DR expression by stimulated pDC remained modest, however, in comparison to unstimulated cDC. Increased SLA-DR expression was also apparent in the cDC1 population following TLR ligation or *A. suum* E/S treatment, although differences were relatively small and not statistically significant in comparison to the high baseline level.

### *Ascaris* E/S strongly enhances CD80/86 expression by porcine cDC2, but powerfully inhibits upregulation of CD80/86 by cDC1 following TLR ligation

Next to antigen presentation, the expression of costimulatory molecules is required for the full activation of naïve T cells. We hence determined the expression of the costimulatory B7 molecules CD80/86 using recombinant human CD152 (CTLA-4) which was shown earlier to cross-react with porcine CD80/86 (23). When unstimulated, 55 ± 12% of cDC1 and 31 ± 23% of cDC2 expressed high levels of CD80/86, compared to 6.5 ± 8.1% of pDC (**Figures 2E, F**). However, following TLR ligation, expression of CD80/86 was greatly upregulated by all DC subsets (cDC1 = 97 ± 2.0%, cDC2 = 82 ± 19%, pDC = 84 ± 13% CD80/86 high). Despite the consistency of response across all subsets to TLR ligation, responses to stimulation with *Ascaris* E/S or combined TLR ligand and E/S stimulation differed strongly between DC subsets. cDC1 exposed to *Ascaris* E/S did not upregulate CD80/86, but E/S strongly inhibited the TLR-driven upregulated of costimulatory molecules. In contrast, cDC2 greatly increased expression of CD80/86 to similar levels as seen after TLR ligation, and uniformly expressed high levels of B7 molecules when exposed to both types of stimuli. On pDC, E/S products led to a modest rise in CD80/86 expression, but partially repressed the strong upregulation induced by TLR ligation.

As seen in **Figure 2B**, cDC1 viability was greatly enhanced, as was expression of CD80/86 when the cells were exposed to TLR ligands. Both of these effects were highly suppressed by the addition of *Ascaris* E/S. Hence, a culminative effect occurs, resulting in a much larger difference in the number of live, CD80/86 high cDC1 following TLR and E/S treatment compared to TLR ligation alone. Approximately 18 ± 9.8% of total singlets were live CD80/86 high cells within the unstimulated cDC1 culture, which was increased to 73 ± 7.3% following TLR ligation, but suppressed to 32 ± 12% by the addition of E/S (**Supplementary Figure S1**). Viability of, and SLA-DR and CD80/86 expression by porcine monocytes were also assessed, but both viability and expression levels were highly heterogenous between pigs, and not significantly modulated by TLR ligation or E/S exposure (**Supplementary Figures S2A–C**).

Taken together, irrespective of exposure to helminth E/S or microbial ligands, porcine cDC exhibit high potential for SLA-DR mediated antigen presentation. Interestingly, the cDC2 subset, accounting for the majority of cDC in peripheral blood, was highly responsive to stimulation with E/S in terms of CD80/86 upregulation. We therefore speculate that cDC2 may be an important player in the activation of the adaptive T cell response in the context of *Ascaris* infection. In contrast, pDC did not significantly upregulate B7 molecules when stimulated with E/S alone, although we did observe a small but significant increase SLA-DR expression. However, this upregulation of SLA-DR occurred in parallel with a significant reduction in pDC viability. Therefore, the total number of SLA-DR expressing pDC was not significantly altered (**Supplementary Figure S1A**). cDC1 displayed no changes in SLA-DR or CD80/86 expression in response to E/S stimulation alone.

### Production of TNF-α by pDC following TLR ligation is suppressed by the addition of *Ascaris* E/S

A superior capacity for cytokine production by porcine pDC compared to cDC has been previously demonstrated by others (21) and by us (20). To elucidate the role of pDC in cytokine production during helminth-driven responses, we collected supernatants from cultured pDC after 24 hours, and determined concentrations of IL-12p40, TNF-α, IL-4 and IL-10 by ELISA.

**Figure 3:**
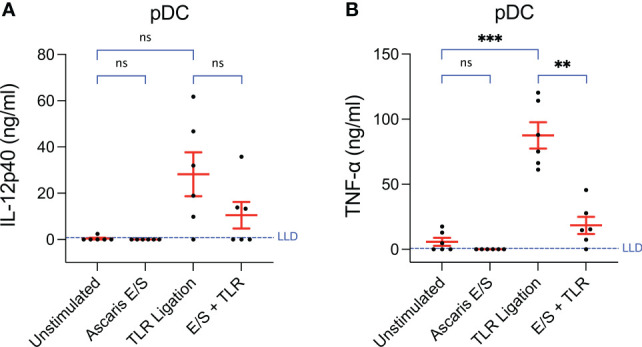
Production of TNF-α by pDC following TLR ligation is suppressed by the addition of *Ascaris* E/S. Concentrations of **(A)** IL-12p40 and **(B)** TNF-α measured by ELISA in the supernatants of pDC incubated at a density of 1x10^6^/ml for 24h. Cells were either unstimulated, or stimulated with *Ascaris* E/S, TLR ligands (100 ng/ml LPS, 500 ng/ml poly(I:C) and 300 ng/ml R848), or E/S and TLR ligands in combination. Supernatants were diluted 1:10 in cIMDM prior to IL-12p40 quantification and 1:20 prior to TNF-α quantification. Lower limits of detection (LLD) – IL-12p40: 0.781 ng/ml, TNF-α: 0.626 ng/ml. IL-4 and IL-10 were also measured but concentrations were undetectable after all treatments. Asterisks indicate statistical significance by paired t test with Bonferroni correction; **p ≤ 0.0033, ***p ≤ 0.00033. ns, not significant.

Unstimulated pDC secreted very low levels of TNF-α (mean = 5.8 ng/ml) and undetectable levels of IL-12p40 (**Figures 3A, B**). Following TLR ligation, the mean quantities of both cytokines produced were very high (IL-12p40 = 28 ± 23 ng/ml, TNF-α = 88 ± 25 ng/ml). For TNF-α, this was statistically significant, however a trend towards increased in IL-12p40 production did not reach statistical significance as the magnitude of the responses varied greatly between individual pigs. Interestingly, when pDC were stimulated with both *A. suum* E/S and TLR ligation simultaneously, the amounts of IL-12p40 and TNF-α produced were much lower (IL-12p70: mean = 10 ± 14 ng/ml; TNF-α: mean = 18 ± 16 ng/ml) than after TLR ligation alone. In the case of TNF-α, this reduction was statistically significant. Neither IL-4 nor IL-10 were detectable under any conditions. Hence, the addition of *Ascaris* E/S to TLR-ligated pDC resulted in reduced production of the type-1 cytokines IL-12p40 and TNF-α, while production of type-2 associated IL-4 and IL-10 remain undetectably low.

In parallel we examined cytokine production by monocytes (**Supplementary Figure S3**) and found TNF-α production following TLR ligation to be modulated by *A. suum* E/S exposure in a similar pattern to that observed in pDC. No upregulation of TNF-α was observed following *Ascaris* E/S exposure alone, but TNF-α production was increased slightly following TLR ligation, with this increase being abolished when cells were treated with TLR ligation and *A. suum* E/S in combination. However, concentrations of TNF-α quantified under all conditions were low, with a mean of only 1.5 ± 0.78 ng/ml observed in the TLR-stimulated monocyte cultures in comparison to 88 ± 25 ng/ml for TLR-stimulated pDC. Interestingly, monocytes did not respond to TLR ligation with IL-12p40 production but did significantly upregulate IL-12p40 following stimulation with *Ascaris* E/S alone, although only to a very low mean concentration of 0.46 ± 0.23 ng/ml. For comparison, TLR ligated pDC cultures contained 28 ± 23 ng/ml IL-12p40. Like pDC, monocytes did not produce detectable levels of IL-4 or IL-10 under any treatment conditions.

### *Ascaris* E/S suppresses IFN-γ production by NK cells following IL-12 and IL-18 stimulation

DC-NK cell cross-talk plays a vital role in the amplification of Th1 polarization signals (17). It has been described in mice that IL-12 and IL-18 produced by DC synergistically induce IFN-γ production by NK cells, with IL-18 driving upregulation of the IL-12 receptor, and IL-12 initiating IFN-γ release (18). Likewise, we have previously reported that IFN-γ production by porcine NK cells is initiated following combined stimulation with IL-12p70 and IL-18, but not stimulation with either cytokine individually (20). NK cell-derived IFN-γ contributes to enhanced IL-12 production by DC (18), thus constituting a positive feedback mechanism, as well as enhancing polarization of naïve CD4+ T cells towards a Th1 phenotype (17). Since we observed a small reduction in IL-12 production by pDC following the addition of *Ascaris* E/S, we investigated whether innate IFN-γ production by NK cells may also be modulated by E/S exposure.

**Figure 4:**
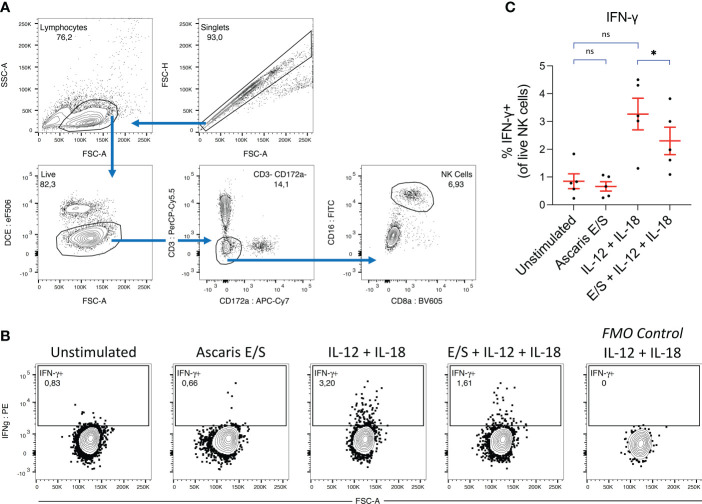
*Ascaris* E/S suppresses IFN-γ production by NK cells following IL-12 and IL-18 stimulation. **(A)** Gating strategy used to identify NK cells in porcine PBMC. Cell aggregates were excluded and lymphocytes were selected based on size (FSC-A) and granularity (SSC-A), before live cells were identified using a fixable viability dye. CD3+ and CD172a+ cells were excluded, and from the remaining cells, a distinct CD16+ CD8 low NK cell population was identified. **(B)** Representative intracellular IFN-γ staining of NK cells from PBMC incubated for 12h with brefeldin A, either unstimulated or stimulated with Ascaris E/S, IL-12p70 + IL-18, or E/S + IL-12p70 + IL-18. Also included is an FMO control sample stimulated with IL-12p70 + IL-18, but not labelled with anti-IFN-γ antibody (right). **(C)** Percentages of IFN-γ+ cells within the NK cell population identified as described, within PBMC samples stimulated with the treatments outlined above. Each dot represents cells from different pig, n = 5. Asterisks indicate statistical significance by paired t test with Bonferroni correction; * p ≤ 0.017. ns, not significant.

Whole porcine PBMC were stimulated with IL-12p70 and IL-18, and/or *Ascaris* E/S for 12 hours. Following incubation, cells were labelled and NK cells were identified by flow cytometry using the gating strategy indicated in **Figure 4A**. NK cells exposed to each treatment were analyzed for intracellular IFN-γ (**Figures 4B, C**). We observed that stimulation with *Ascaris* E/S alone had no impact on IFN-γ production by NK cells compared to the unstimulated control. However interestingly, the addition of E/S to IL-12p70 and IL-18 stimulated PBMC did significantly reduce the number of IFN-γ+ NK cells within PBMC. Therefore, we find that *Ascaris* E/S appears to suppress innate Th1-inducing signals at multiple levels. Not only does it reduce production of IL-12 by TLR-stimulated pDC, but also significantly suppressed production of IFN-γ by NK cells in response to IL-12 and IL-18 stimulation.

To exclude the possibility of direct degradation of IL-12 by *A. suum* E/S, different concentrations of recombinant IL-12p70 (0, 50, 100, 200 ng/ml) were incubated together with different concentrations of *Ascaris* E/S (0, 50, 100, 200 μg/ml). After 24 hours, IL-12 concentrations were measured by ELISA. No changes in IL-12 concentration was detected (**Supplementary Figure S4**).

### *Ascaris* E/S prevents SEB-driven T cell activation by TLR-activated DC

**Figure 5:**
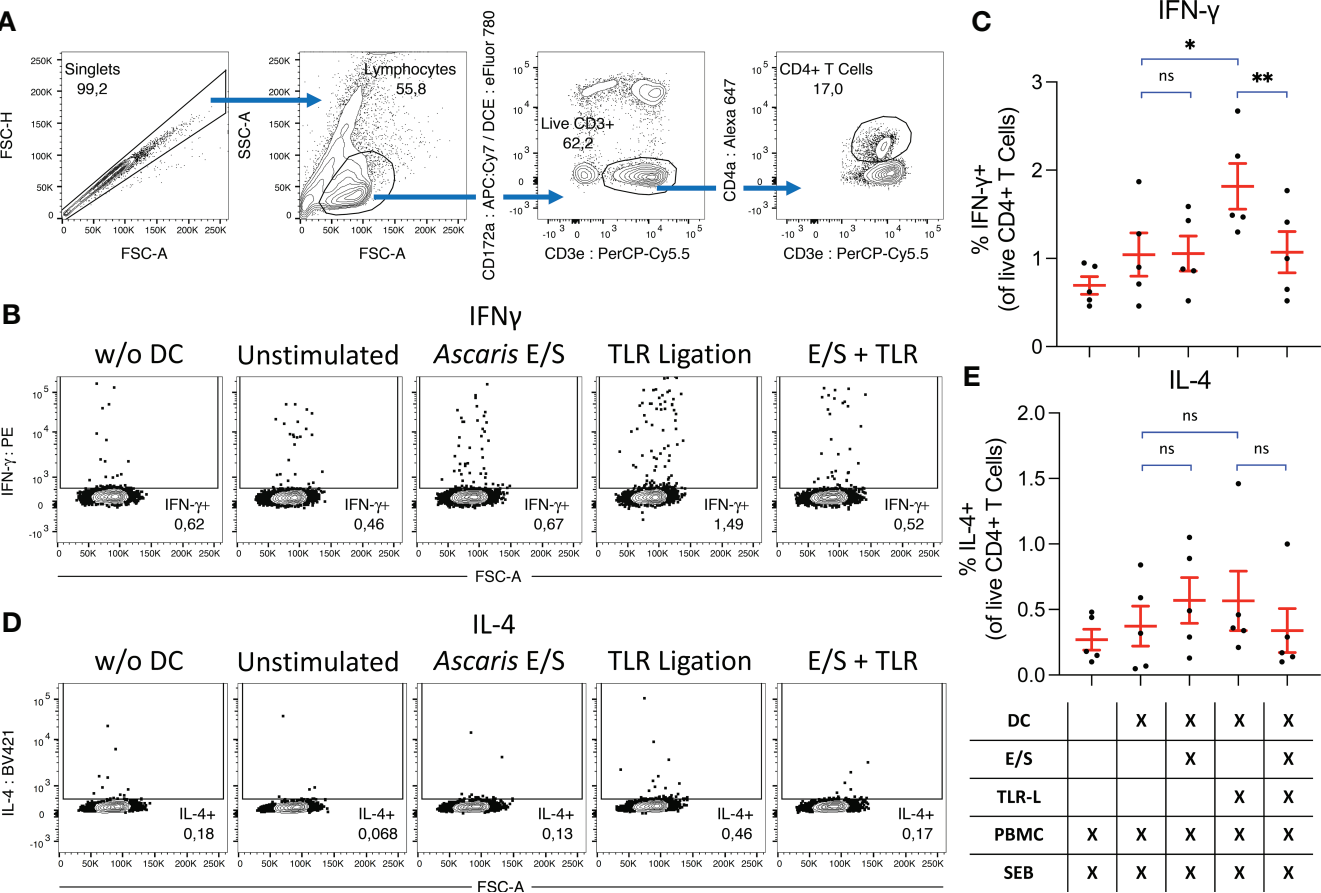
*Ascaris* E/S prevents SEB-driven T cell activation by TLR-activated DC. **(A)** Representative gating strategy used to identify CD4+ T helper cells. Cells aggregates were excluded, and lymphocytes selected on the basis of size and granularity. CD172a- CD3e+ T cells were selected, and within this population CD4+ T helper cells were identified. During previous DC subset sorting, pDC were labelled with CD4a-PerCP-Cy5.5, and these cells appear as a false “CD172a+ CD3e+” population due to re-use of the PerCP-Cy5.5 fluorophore. Since all DC subsets are CD172a+, we can be sure that the CD172a- population has not previously been exposed to labelling and thus the CD172a- CD3e+ population constitutes a true T cell population. **(B)** Representative intracellular IFN-γ staining of CD4+ T cells from PBMC cultured without DC (left), or co-cultured with DC which were unstimulated, or stimulated with *Ascaris* E/S, TLR ligation, or E/S + TLR ligation. **(C)** Percentage IFN-γ+ CD4+ T cells identified under each treatment condition. Treatments are displayed in the grid below. Each dot represents cells from a different pig, n=2. **(D)** Intracellular IL-4 staining of CD4+ T cells from PBMC cultured without DC (left), or co-cultured with DC which were unstimulated, or stimulated with *Ascaris* E/S, TLR ligation, or E/S + TLR ligation. **(E)** Percentage IL-4+ CD4+ T cells identified under each treatment condition. Treatments are displayed in the grid below. Each dot represents cells from different pig, n=5. Asterisks indicate statistical significance by paired t test with Bonferroni correction; * p ≤ 0.017, ** p ≤ 0.0033. ns, not significant.

Finally, we investigated the impact of *A. suum* E/S on the capacity of DC to activate T helper cells. To that end, we combined cDC1, cDC2 and pDC in their natural proportions (**Figure 1C**), and treated them with *Ascaris* E/S and/or TLR ligation as described above. After 18 hours, autologous, unsorted PBMC were added at a 1:1 ratio for a further 18 hours. To stimulate T cells and monitor their activity, the bacterial superantigen SEB was added. Cytokine excretion was blocked during the last 12 hours of culture and IFN-γ and IL-4 production by CD4+ T cells was quantified by FACS (**Figures 5A, E**).

When co-cultured DC and autologous unsorted PBMC were treated with SEB alone, we observed IFN-γ production by 1.0 ± 0.55%, and IL-4 production by 0.37 ± 0.34% of CD4+ T cells. SEB-driven IFN-γ, but not IL-4, production by CD4+ T cells was significantly enhanced when co-cultured DC were treated with TLR ligands. In contrast, neither IFN-γ nor IL-4 production was upregulated following stimulation with *Ascaris* E/S alone. Further, simultaneous treatment of DC with *A. suum* E/S and TLR ligands completely prevented the increased IFN-γ production by co-cultured T helper cells seen in co-cultures with TLR-activated DC.

## Discussion

During parasite infection, DC are essential for initiating and influencing the direction of the subsequent T cell response. As such, host DC are a common target for modulation by helminths, and there are many examples of DC manipulation by helminth E/S (9). Murine bmDC treated with E/S from *Nippostronglyus brasiliensis, Heligmosomoides polygyrus* (28) or *Fasciola hepatica* (29) all induce Th2 polarization, mediated at least in part through suppression of Th1-inducing signals such as IL-12 production. The same effect has been reported for murine bmDC and human monocyte-derived DC (moDC) treated with *Schistosoma mansoni* soluble egg antigen. In the case of *S. mansoni*, this immunomodulation of DC is driven by a single glycoprotein, ω-1, which suppresses IL-12 and co-stimulatory molecule expression through degradation of intracellular RNA (30). In contrast, murine bmDC treated with *Echinococcus multilocularis* E/S (31) and human moDC co-cultured with live *Brugia malayi* (32) have been shown to undergo apoptosis, highlighting that helminths can incapacitate host DC *via* diverse mechanisms.

Here we investigated the impact of *Ascaris* E/S on the functions of porcine DC, hence examining potential modulation of immune cells from the parasite’s definitive host species. Previous studies have examined immunomodulation of murine bmDC by *A. suum* extract (13) and pseudocoelomic body fluid (ABF) from the closely-related *A. lumbricoides* (12), as well as of human moDC by *A. suum* ABF (14, 15). While this data provided hints to possible modulatory effects of *Ascaris* exposure on DC phenotypes *in vivo*, several limitations exist. Firstly, proteomic analysis by Chehayeb et al. (2014) has demonstrated distinct differences in the composition of *A. suum* E/S compared to ABF and uterine fluid (33). Therefore, to more accurately represent DC exposure to *A. suum* during *in vivo* infection, it may be beneficial to treat DC with *A. suum* E/S released naturally from live, adult worms. Secondly, the phenotypes of *in vitro*-generated DC following *Ascaris* exposure may not be representative of *in vivo* DC subset diversity. moDC differentiate from monocytes *in vivo* only at sites of inflammation, and so are not present during steady-state conditions (10), while studies based on murine bmDC may be afflicted by host-parasite mismatch. Thus, studies focusing on *A. suum* E/S and their impact on *ex vivo bona fide* DC more likely represent a system suited for evaluating the phenotypic consequences of *A. suum* exposure on DC during infection.

Our data demonstrates that porcine peripheral blood cDC constitutively express high levels of SLA-DR, a component of porcine MHC-II. In contrast, we found unstimulated peripheral blood pDC to express very low levels of SLA-DR in the pig, although SLA-DR expression was moderately upregulated following stimulation with TLR ligands or *A. suum* E/S. These results correspond to previous findings by Auray et al. (2016) and Edwards et al. (2017) which report very high SLA-DQ and SLA-DR expression by cultured porcine cDC respectively (21, 27). Similar to our findings, Edwards et al. (2017) reported low expression of SLA-DR by unstimulated porcine pDC, whereas Auray et al. (2016) reported heterogenous expression of SLA-DQ (21, 27). This in contrast to murine splenic pDC (34, 35) and human peripheral blood pDC (24), which constitutively express high levels of MHC-II, albeit at a slightly lower level than cDC. To our knowledge, upregulation of MHC-II by porcine pDC following stimulation has not been previously reported. However, this result is in line with findings from murine splenic pDC, which have been demonstrated to upregulate MHC-II following TLR 9 ligation with CpG or infection with influenza (34). Interestingly, Young et al. (2008) demonstrated that while murine splenic cDC express higher levels of MHC-II and are more efficient at inducing T cell proliferation, pDC much more quickly synthesize and turnover MHC-II-peptide complexes (34). This rapid turnover of MHC-II complexes likely endows pDC with the capacity to continuously present new antigen at the site of infection, in contrast to cDC which provide superior antigenic memory (34, 35).

We also observed substantial subset-specific differences in expression of the important co-stimulatory molecules CD80/86 following *Ascaris* E/S exposure. cDC1, cDC2 and pDC all strongly upregulated CD80/86 following the combined ligation of TLRs 3, 4, 7 and 8 *via* poly(I:C), LPS and R848 stimulation. However, following stimulation with E/S alone, only cDC2 responded by strongly upregulating CD80/86. We previously reported a similar upregulation of CD80/86 by cDC2 but not cDC1 following stimulation with LPS and R848 (20). Since *Ascaris* E/S contains LPS (1 ng endotoxin per μg E/S), it seems likely the upregulation of CD80/86 observed in the cDC2 subset following E/S stimulation partially depends on LPS contaminations.

Strikingly, when cDC1 were stimulated with E/S in addition to TLR ligation, CD80/86 expression was strongly suppressed. This affect was also apparent for pDC to a lesser extent. Similarly, Favoretto et al. (2014) demonstrated that high molecular weight components (P1) isolated from *A. suum* extract similarly suppressed upregulation of CD80 and CD86 by murine bmDC following ligation of TLR 1/2, 3 or 4 (13). Expression of the co-stimulatory molecule CD40 and MHC-II were also suppressed in this model. Suppression of antigen presentation is broadly in line with our findings, although we did not observe a reduction of SLA-DR expression in our system. Favoretto et al. (2017) later showed that when bmDC were pre-incubated with mannan, a ligand of several C-type lectins (36), the inhibitory effects of P1 on co-stimulatory molecule and MHC-II expression following LPS stimulation were blocked (37). This effect was partially recapitulated by antibody blockade of the C-type lectin DC-SIGN or mannose receptors, suggesting P1 suppresses antigen presentation *via* C-type lectin signaling. It is tempting to speculate that the phenotype seen in our studies suggests a comparable mode of action for *Ascaris* E/S-driven modulation of B7 expression by porcine DC1 and pDC. Further research is needed to evaluate this hypothesis.

In addition to suppression of CD80/86 expression, we also observed a strong suppression of TLR-driven TNF-α production by pDC following treatment with E/S in addition to TLR ligation. Mean IL-12p40 production was also suppressed, although responses were highly heterogenous between individual pigs. These findings are again similar to those by Favoretto et al. (2014), who demonstrated reduced production of IL-12p70 by murine bmDC following TLR stimulation when P1 was added (13). Likewise, Midttun et al. (2018) demonstrated suppressed upregulation of IL-12 and TNF-α by human moDC following LPS treatment combined with exposure to *A. suum* ABF, compared to LPS treatment alone (14). In contrast, porcine pDC did not respond with IL-12 production following *A. suum* E/S stimulation alone. Auray et al. (2016) previously reported that IL-4 and IL-10 were not produced in significant quantities by any porcine DC subset following stimulation with a wide range of TLR ligands (21). In line with these findings, we did not observe production of either cytokine by pDC following TLR ligation or *A. suum* E/S stimulation. Conversely, Midttun et al. (2018) reported IL-10 production by human moDC, and Favoretto et al. (2014) described IL-10 production by murine bmDC following LPS stimulation (13, 14).

It is not possible from our data to distinguish between changes in the number of cytokine-producing pDC and changes in the quantity of cytokine production on a per-cell basis. However, the suppression of cytokine production observed when pDC were treated with *A. suum* E/S in addition to TLR ligation was likely mediated predominantly by cytotoxic properties of E/S. It has been previously reported that murine splenic DC exhibit a relatively short lifespan *in vitro* (26), and we observed similarly low viability of unstimulated porcine peripheral blood DC, especially pDC. Strikingly however, we observed large and subset-specific fluctuations in survival following stimulation. Interestingly, when exposed to *Ascaris* E/S, viability of cDC was not reduced, whereas the more short-lived pDC were almost completely depleted within 24 hours of E/S exposure. This therefore indicates pDC-specific cytotoxicity of *A. suum* E/S. Indeed, when pDC were treated with *A. suum* E/S in addition to TLR ligation, this loss of viability was less drastic but significant – dropping from 22% following TLR ligation to 12% with the addition of E/S. Consequently, lower cytokine concentrations in the supernatant would be expected due to the presence of fewer live, cytokine-producing cells. Interestingly, *A. suum* E/S did not reduce cDC1 viability per se, but prevented the prolonged survival of cDC1 seen after TLR stimulation. This impairment of cDC1 survival together with repression of TLR-mediated B7 upregulation may therefore result in a culminative effect. Further work should assess changes in the frequencies and phenotypes of individual DC subsets in gut-associated lymphoid tissue during *Ascaris* infection.

DC-NK cell cross-talk is important for enhancing Th1 polarization signals, since DC are a major source of both IL-12 and IL-18 which synergistically promote IFN-γ and TNF production by NK cells. In turn, NK cell-derived IFN-γ and TNF enhance IL-12 production by DC, creating a positive feedback loop (17, 18). We know from previous experiments that treatment of porcine NK cells with recombinant IL-12p70 and IL-18 induces innate IFN-γ production (20). Therefore, we utilized this system to investigate whether IFN-γ production by porcine NK cells was directly modulated by *A. suum* E/S. We demonstrate that treatment of PBMC with E/S did not induce IFN-γ production by NK cells, but treatment with E/S in addition to IL-12p70 and IL-18 significantly suppressed IFN-γ production. This result is striking as it highlights the capacity of *A. suum* E/S to suppress IFN-γ production not only *via* manipulation of host DC but *via* diverse pathways.

We further investigated the consequences of DC modulation by *A. suum* E/S on their capacity to activate T cell responses by co-culturing DC treated with E/S and/or TLR ligation with autologous unsorted PBMC. SEB was added during co-culture to induce non-specific T cell activation by cross-linking CD80/86 on DC with CD28 and the T cell receptor expressed by T cells (38). This approach enables antigen-independent activation of T cells, while remaining sensitive to differences in CD80/86 expression by DC observed between treatment groups. All three sorted *bona fide* DC subpopulations were combined in their naturally occurring ratios prior to treatment, to enable interaction of T cells with both antigen-presenting cDC and pDC-derived cytokines. FACS analysis was performed to identify CD4+ T helper cells, and intracellular staining was used to quantify production of IFN-γ and IL-4, associated with Th1 and Th2 responses respectively. We previously reported that stimulation of porcine PBMC with SEB induced activation of 0.21 to 1.07% of CD4+ T cells, identified by expression of the costimulatory molecule CD154 (39). The same paper also described co-expression of CD154 and IFN-γ. Hence, the percentage of IFN-γ+ CD4+ T cells detected in our assay following stimulation with SEB alone (1.0 ± 0.55%) falls within the expected range. We found that when DC were pretreated with TLR ligation, IFN-γ production by CD4+ T cells was greatly enhanced. However, when DC were pre-treated with TLR ligation and *A. suum* E/S in combination, this enhancement was completely inhibited. Very low levels of IL-4 production by CD4+ T cells after SEB stimulation were not altered when co-cultured DC were pre-treated with either TLR ligation or *A. suum* E/S.

SEB-mediated activation of CD4+ T cells relies on interaction between the T cell receptor and CD28 expressed by T cells, and MHC-II and B7 molecules expressed by an antigen-presenting cell. We reported significantly suppressed expression of CD80/86 by TLR-stimulated cDC1 and pDC upon co-exposure to *A. suum* E/S, and significantly reduced viability of cDC1. It therefore seems likely that the combination of these effects resulted in reduced IFN-γ production by CD4+ T cells in our co-culture assays. It should be noted that we did not wash DC prior to the addition of autologous PBMC, to avoid the removal of DC-derived cytokines. Therefore, it is also possible that differences in cytokine environment play a role in the different IFN-γ responses by CD4+ T cells. Further, we cannot rule out the possibility that E/S acts directly on T cells to suppress IFN-γ production, similarly to our findings in NK cells. Interestingly, reduced IFN-γ production by human CD4+ T cells upon PMA/ionomycin stimulation following co-culture with human moDC treated with *A. suum* ABF and LPS in comparison to LPS alone was previously reported (14). Of note, this study used DC which were washed after ABF pre-treatment, prior to co-culture with isolated CD4+ T cells, suggesting a B7-dependent mechanism.

**Figure 6:**
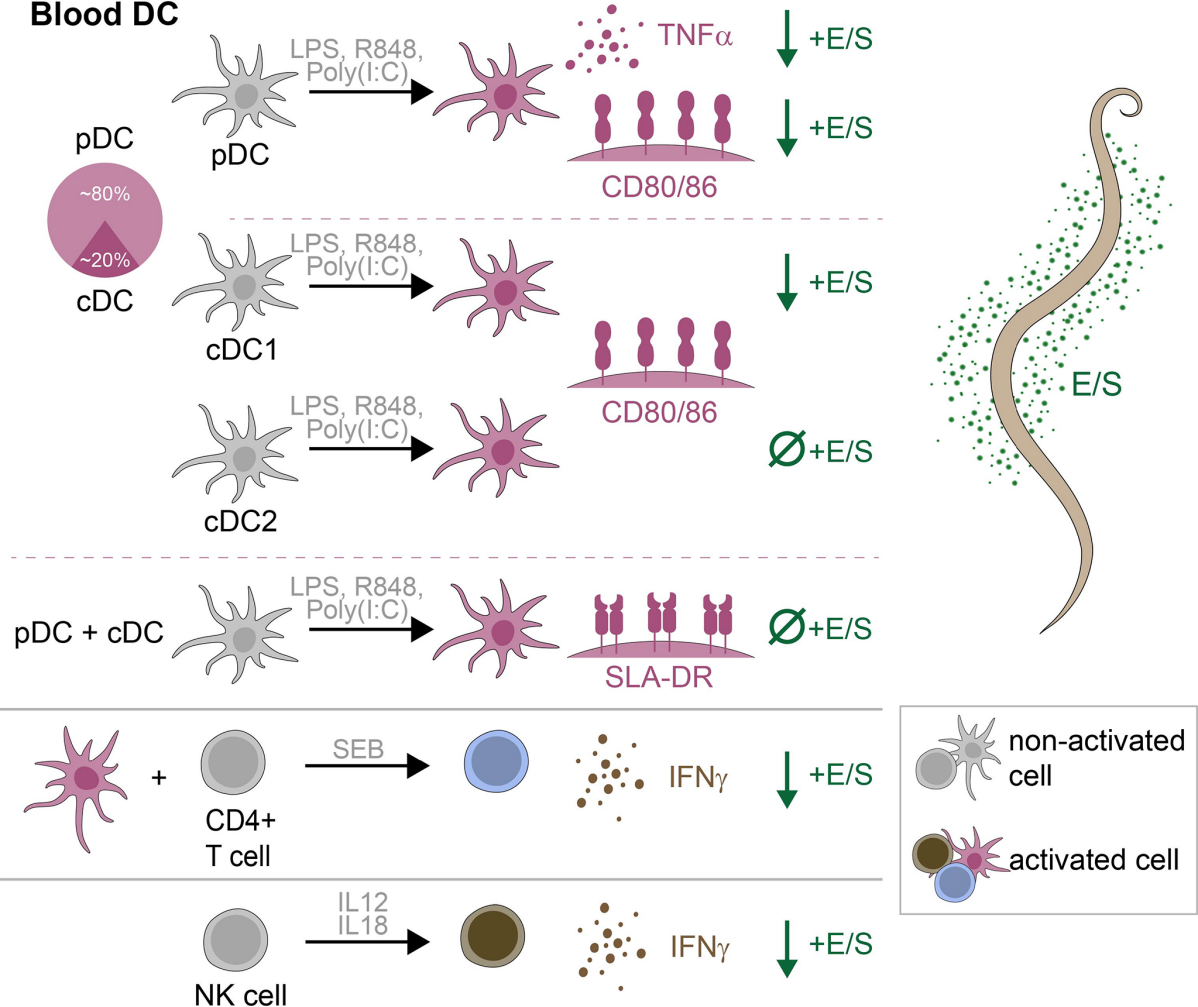
Graphical summary of key findings. Within porcine peripheral blood, DC consisted of approximately 80% pDC and 20% cDC. Following stimulation with LPS, R848 and poly(I:C), TNF-α production by pDC, and CD80/86 expression by pDC, cDC1 and cDC2 were significantly upregulated. With the further addition of *A. suum* E/S, TNF-α production by pDC, and CD80/86 expression by pDC and cDC1, were significantly reduced. The addition of *A. suum* E/S did not reduce expression of CD80/86 by TLR-ligated cDC2, or expression of SLA-DR by any DC subset. When SEB-treated PBMC were co-cultured with autologous TLR-ligand pre-treated DC, CD4+ T cells significantly upregulated IFN-γ production. When DC were pre-treated with E/S in addition to TLR-ligation, this IFN-γ production was significantly reduced. Finally, NK cells treated with recombinant porcine IL-12p70 and IL-18 significantly upregulated IFN-γ production, which was significantly reduced when cell were treated with E/S in addition to cytokine stimulation.

The same authors also analyzed jejunal mucosa samples from pigs chronically infected with *A. suum* by microarray and qPCR (14). Relative to uninfected pigs, infected pigs exhibited a downregulation of many pro-inflammatory genes, including those encoding MHCII, CD80, CD86, and IFN-γ. Here we demonstrate that *A. suum* E/S can modulate expression of MHCII and CD80/86 by porcine *bona fide* DC *ex vivo*, as well as alter survival of porcine DC, thus affecting both functionality and availability of antigen presenting cells. However, the DC population consists of diverse subsets with distinct ontogenies and phenotypes, which appear to be differentially modulated by *A. suum* E/S. We demonstrate that *A. suum* E/S-treated DC are unable to induce IFN-γ production by co-cultured CD4+ T cells, and describe an independent suppression of IFN-γ production by NK cells following *A. suum* E/S exposure. Therefore, *A. suum* E/S likely plays an important role in inhibiting Th1-driving signals during *A. suum* infection in its natural host. Key findings are summarized graphically in **Figure 6**.

## Data availability statement

The original contributions presented in the study are included in the article/**Supplementary Material**. Further inquiries can be directed to the corresponding author.

## Author contributions

Conceptualization – BH and SH. methodology – BH, FE, SR and SH. validation – BH. formal analysis – BH. investigation – BH, LB and AK. resources – SH. data curation – BH. writing (original draft preparation) – BH. writing (review and editing). BH, LB, AK, FE, SR and SH. visualization – BH. supervision – SH. project administration – BH. funding acquisition – SH. All authors have read and agreed to the published version of the manuscript.

## Funding

We acknowledge funding by the German Research Foundation (DFG) to SH within the projects GRK 2046-project B4 and DFG HA 2542/11-1. We acknowledge support by the Open Access Publication Initiative of Freie Universität Berlin.

## Acknowledgments

Beate Anders (Institute of Immunology, Freie Universität Berlin, Berlin, Germany) for blood sample collection and technical assistance with E/S preparation; Yvonne Weber (Institute of Immunology, Freie Universität Berlin, Berlin, Germany) for technical assistance with MACS and FACS; Marion Müller (Institute of Immunology, Freie Universität Berlin, Berlin, Germany) for technical assistance with LPS quantification and PBMC isolation; Bettina Sonnenburg (Institute of Immunology, Freie Universität Berlin, Berlin, Germany) for technical assistance with PBMC isolation. Phillip Kliem (Fleischerei Lehmann GmbH, Trebbin, Brandenburg, Germany) for regular provision of porcine blood samples. Anne Winkler (Institute of Immunology, Freie Universität Berlin, Berlin, Germany) for creation of the graphical abstract. Roswitha Merle (Institute for Veterinary Epidemiology and Biostatistics, Freie Universität Berlin, Germany) for advice regarding appropriate statistical analyses.

## Conflict of interest

The authors declare that the research was conducted in the absence of any commercial or financial relationships that could be construed as a potential conflict of interest.

## Publisher’s note

All claims expressed in this article are solely those of the authors and do not necessarily represent those of their affiliated organizations, or those of the publisher, the editors and the reviewers. Any product that may be evaluated in this article, or claim that may be made by its manufacturer, is not guaranteed or endorsed by the publisher.
