# Supplementary material for: Ascaris suum excretory/secretory products differentially modulate porcine dendritic cell subsets
Source: Front Immunol. 2022 Nov 10;13:1012717. doi: 10.3389/fimmu.2022.1012717 (PMC9691337; doi:10.3389/fimmu.2022.1012717)
Supplement: Supplementary file 1 [file DataSheet_1.pdf]

Supplementary Material

1 Supplementary Tables

Target	Clone	Reactive Species	Host Species	Isotype	Conjugate	Manufacturer	Product Code	RRID
CD3e	PPT3	Pig	Mouse	IgG1	-	Bio-Rad	MCA5951GA	-
gdTCR1	PGBL22A	Pig	Mouse	IgG1	-	KingFisher	WS0621S-100	-
CD21	BB6-11C9.6	Pig	Mouse	IgG1	-	Biozol	SBA-4530-01	-
IgM	F008-1629	Pig	Mouse	IgG1	-	BD Biosciences	552551	AB_394423
IgG1	X-56	Mouse	Rat	IgG1	MACS MicroBeads	Miltenyi Biotech	130-047-101	AB_244354
CD14	TÜK4	Human (Cross-reactive to pig)	Mouse	IgG2a	MACS MicroBeads	Miltenyi Biotech	130-050-201	AB_2665482
IgG1	RMG1-1	Mouse	Rat	IgG	APC-Cy7	BioLegend	406620	AB_2571947
CD14	MIL2	Pig	Mouse	IgG2b	-	Bio-Rad	MCA1218GA	-
IgG2b	Polyclonal	Mouse	Goat	-	APC-Cy7	Southern Biotech	SBA-1090-19	-
CD172a	74-22-15A	Pig	Mouse	IgG2b	PE	BD Biosciences	561498	AB_10680275
CADM1	3E1	Human (Cross-reactive to pig)	Chicken	IgY	Alexa 647	Biozol	MBL-CM004-A64	-
CD4a	74-12-4	Pig	Mouse	IgG2b	PerCP-Cy5.5	BD Biosciences	561474	AB_10683310
CD80/86	CD152 (fusion protein)	Human (Cross-reactive to pig)	Mouse (Fc region)	IgG2a	-	Ancell	ANC-501-820	-
IgG2a	R19-15	Mouse	Rat	IgG1	Brilliant Violet 605	BD Biosciences	564024	AB_2738549
SLA-DR	2E9/13	Pig	Mouse	IgG2b	FITC	Bio-Rad	MCA2314F	AB_567376
CD172a	BL1H7	Pig	Mouse	IgG1	-	Bio-Rad	MCA2312GA	-
CD8a	76-2-11	Pig	Mouse	IgG2a	-	ThermoFisher	MA528717	AB_2745676
CD3	BB23-8E6-8C8	Pig	Mouse	IgG2a	PerCP-Cy5.5	BD Biosciences	561478	AB_10680273
CD16	G7	Pig	Mouse	IgG1	FITC	Bio-Rad	MCA1971F	AB_2104030
IFNg	P2G10	Pig	Mouse	IgG1	PE	BD Biosciences	559812	AB_397341
CD4a	74-12-4	Pig	Mouse	IgG2b	Alexa 647	BD Biosciences	561472	-
IL-4	MP4-25D2	Human (Cross-reactive to pig)	Rat	IgG1	Brilliant Violet 421	BioLegend	500826	AB_2561679

Supplementary Table 1: Full information of all antibodies used in this project.

Figure 2B - Viability

Cell:	cDC1				cDC2				pDC			
Treatment:	Unstim.	E/S	TLR	E/S + TLR	Unstim.	E/S	TLR	E/S + TLR	Unstim.	E/S	TLR	E/S + TLR
p =	0.1774	0.1858	0.3539	0.5539	0.5745	0.0214	0.0993	0.6141	0.7614	0.1138	0.6602	0.0128

Figure 2D - SLA-DR

Cell:	cDC1				cDC2				pDC			
Treatment:	Unstim.	E/S	TLR	E/S + TLR	Unstim.	E/S	TLR	E/S + TLR	Unstim.	E/S	TLR	E/S + TLR
p =	0.0346	0.1933	0.0047	0.4366	0.0083	0.3527	0.0528	0.0095	0.0047	0.4013	0.7986	0.0387

Figure 2F - CD80/86

Cell:	cDC1				cDC2				pDC			
Treatment:	Unstim.	E/S	TLR	E/S + TLR	Unstim.	E/S	TLR	E/S + TLR	Unstim.	E/S	TLR	E/S + TLR
p =	0.1412	0.1505	0.3100	0.5877	0.4947	0.4549	0.0160	0.6607	0.0134	0.5919	0.0379	0.0345

Figure 3A - IL-12p40

Cell:	pDC			
Treatment:	Unstim.	E/S	TLR	E/S + TLR
p =	0.0001	n/a	0.8960	0.0547

Figure 3B - TNF-α

Cell:	pDC			
Treatment:	Unstim.	E/S	TLR	E/S + TLR
p =	0.0702	n/a	0.3522	0.6658

Figure 4C - IFN-γ

Cell:	NK cells			
Treatment:	Unstim.	E/S	IL-12 + IL-18	E/S + IL-12 + IL-18
p =	0.3464	0.3167	0.4689	0.7688

Figure 5C - IFN-γ

Cell:	CD4+ T cells				
Treatment:	w/o DC	Unstim.	E/S	TLR	E/S + TLR
p =	0.2832	0.7623	0.5397	0.2500	0.6961

Figure 5E - IL-4

Cell:	CD4+ T cells				
Treatment:	w/o DC	Unstim.	E/S	TLR	E/S + TLR
p =	0.1551	0.4801	0.6365	0.0144	0.0130

Supplementary Table 2: Results of Shapiro-Wilk tests conducted for all datasets prior to further statistical analysis. Datasets for which $p \geq 0.05$ (highlighted green) are considered to have passed the normality test.

2 Supplementary Figures

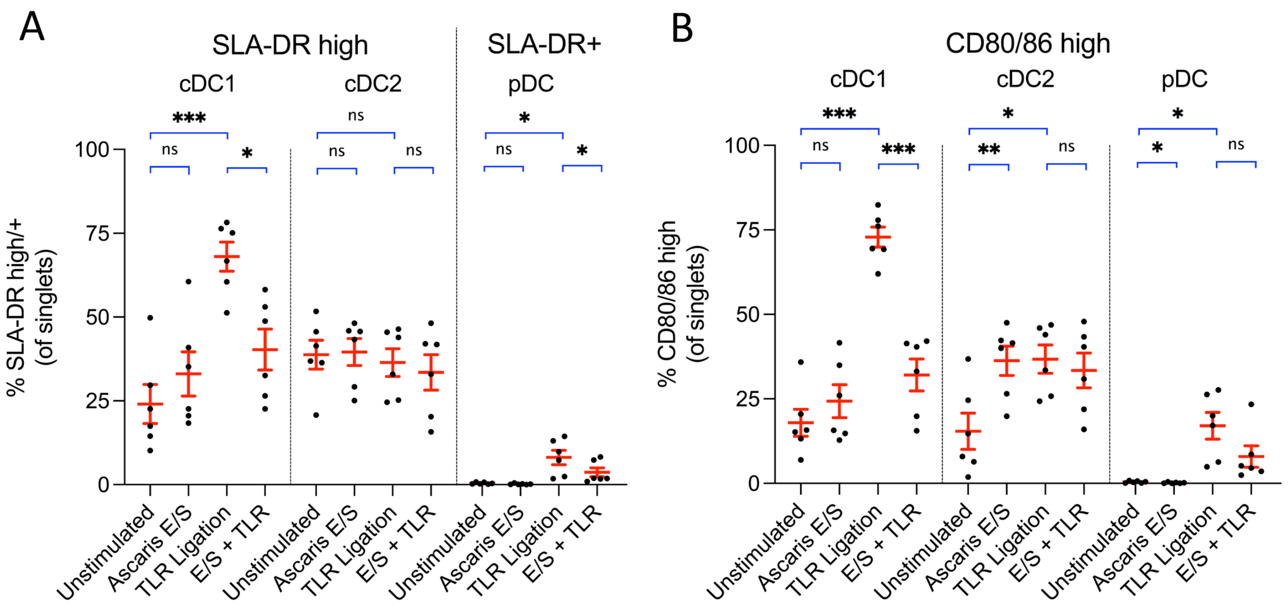

Supplementary Figure 1: (A) Percentages of live SLA-DR high cDC1 and cDC2, and live SLA-DR+ pDC following 24h incubation without stimulation, or stimulation with *Ascaris* E/S, TLR ligation (100 ng/ml LPS, 500 ng/ml poly(I:C) and 300 ng/ml R848), or combined E/S and TLR stimulation, of total singlets. **(B)** Percentages of live CD80/86 high cells for each DC subset and treatment, of total singlets. **(A, B)** Each dot represents a single pig, n = 6. Asterisks indicate statistical significance by paired t test; * p ≤ 0.017, ** p ≤ 0.0033, *** p ≤ 0.00033.

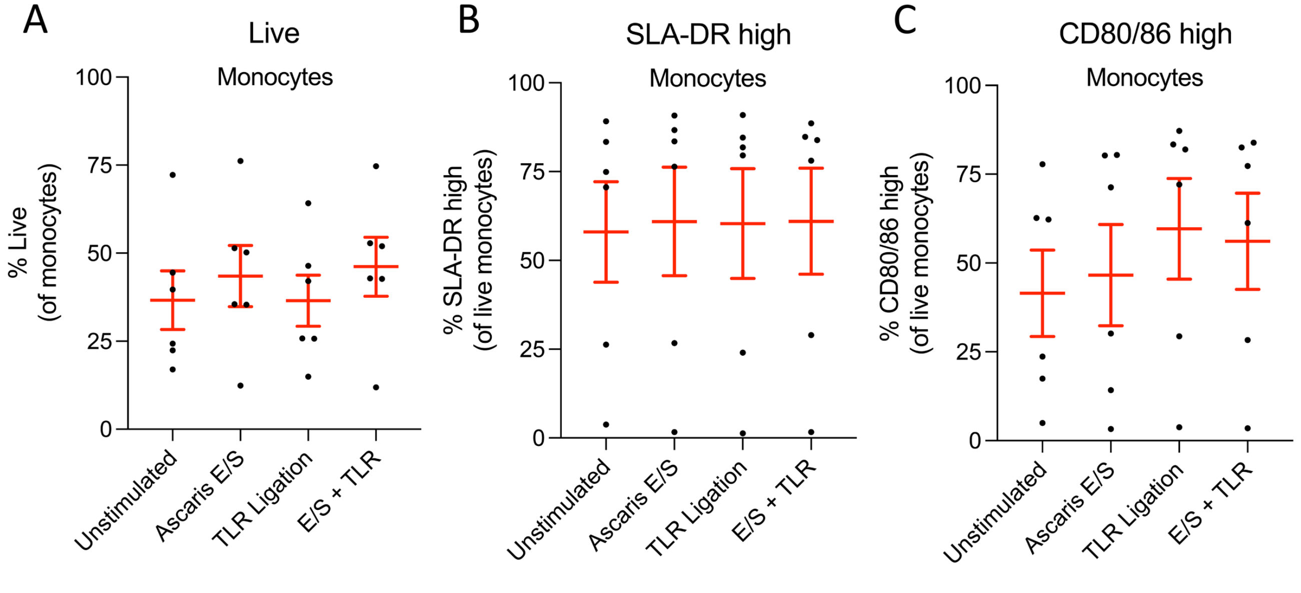

Supplementary Figure 2: (A) Percentages of live monocytes following 24h incubation without stimulation, or stimulation with *Ascaris* E/S, TLR ligation (100 ng/ml LPS, 500 ng/ml poly(I:C) and 300 ng/ml R848), or combined E/S and TLR stimulation. **(B)** Percentages of SLA-DR high

monocytes following each treatment. **(C)** Percentages of CD80/86 high monocytes following each treatment. **(A, B, C)** Each dot represents a single pig, n = 6.

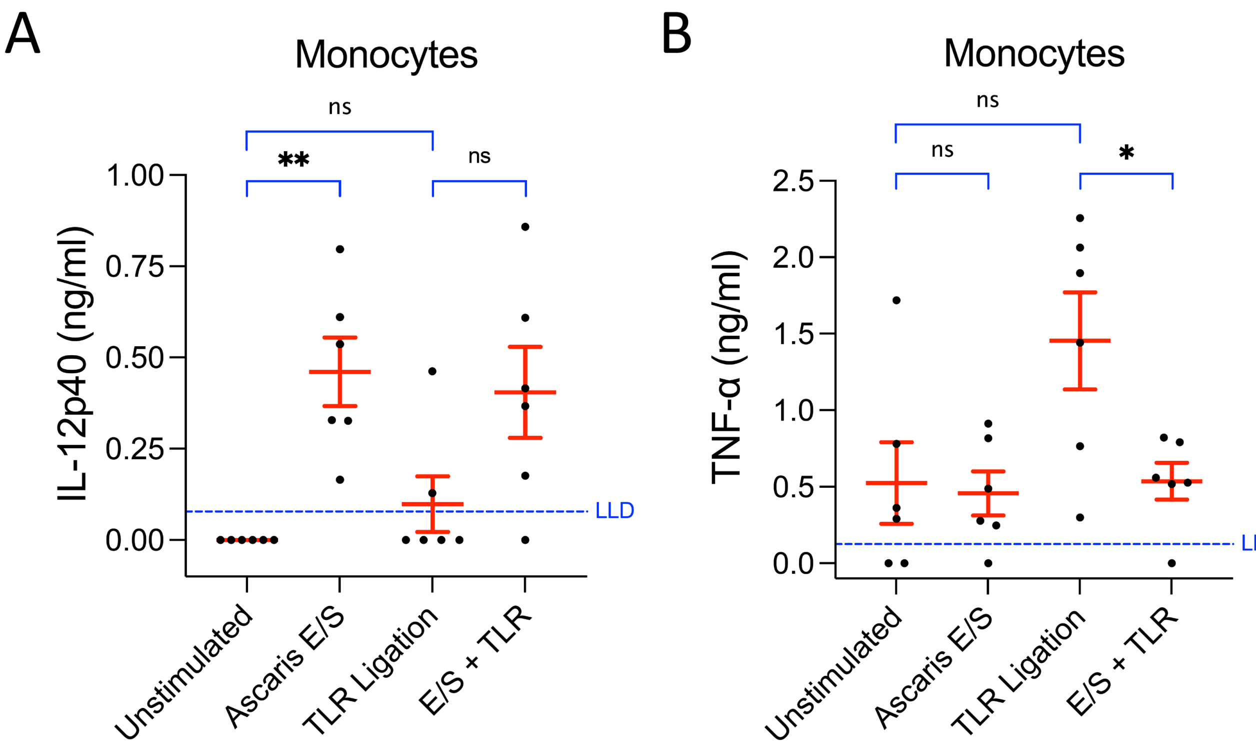

Supplementary Figure 3: Concentrations of **(A)** IL-12p40 and **(B)** TNF-α measured by ELISA in the supernatants of monocytes incubated at a density of $1x10^6$/ml for 24h. Cells were either unstimulated, or stimulated with *Ascaris* E/S, TLR ligands (100 ng/ml LPS, 500 ng/ml poly(I:C) and 300 ng/ml R848), or E/S and TLR ligands in combination. Supernatants were not diluted prior to IL-12p40 quantification, but were diluted 1:4 in cIMDM prior to measurement of TNFa. Lower limits of detection (LLD) – IL-12p40: 0.0781 ng/ml, TNFa: 0.1252 pg/ml. IL-4 and IL-10 were also measured but no concentration changes were detectable after any treatment. Asterisks indicate statistical significance by paired t test; * $p \leq 0.017$, ** $p \leq 0.0033$.

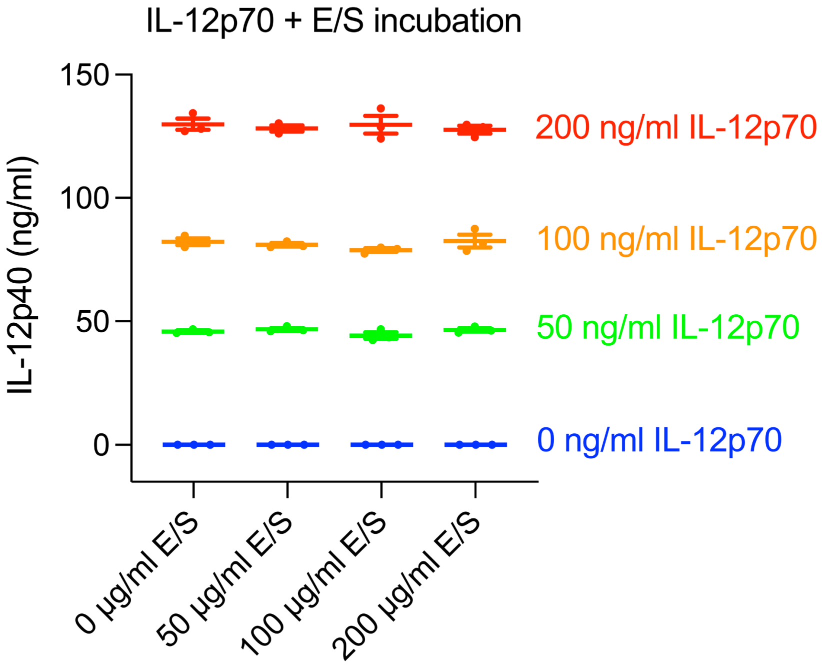

Supplementary Figure 4: Concentrations of IL-12p40 measured by ELISA following co-incubation of recombinant porcine IL-12p70 with different concentrations of *A. suum* E/S for 24h. Different starting concentrations of IL-12p70 are displayed in different colors on overlaid graphs. All samples were diluted 1:20 in cIMDM prior to analysis.

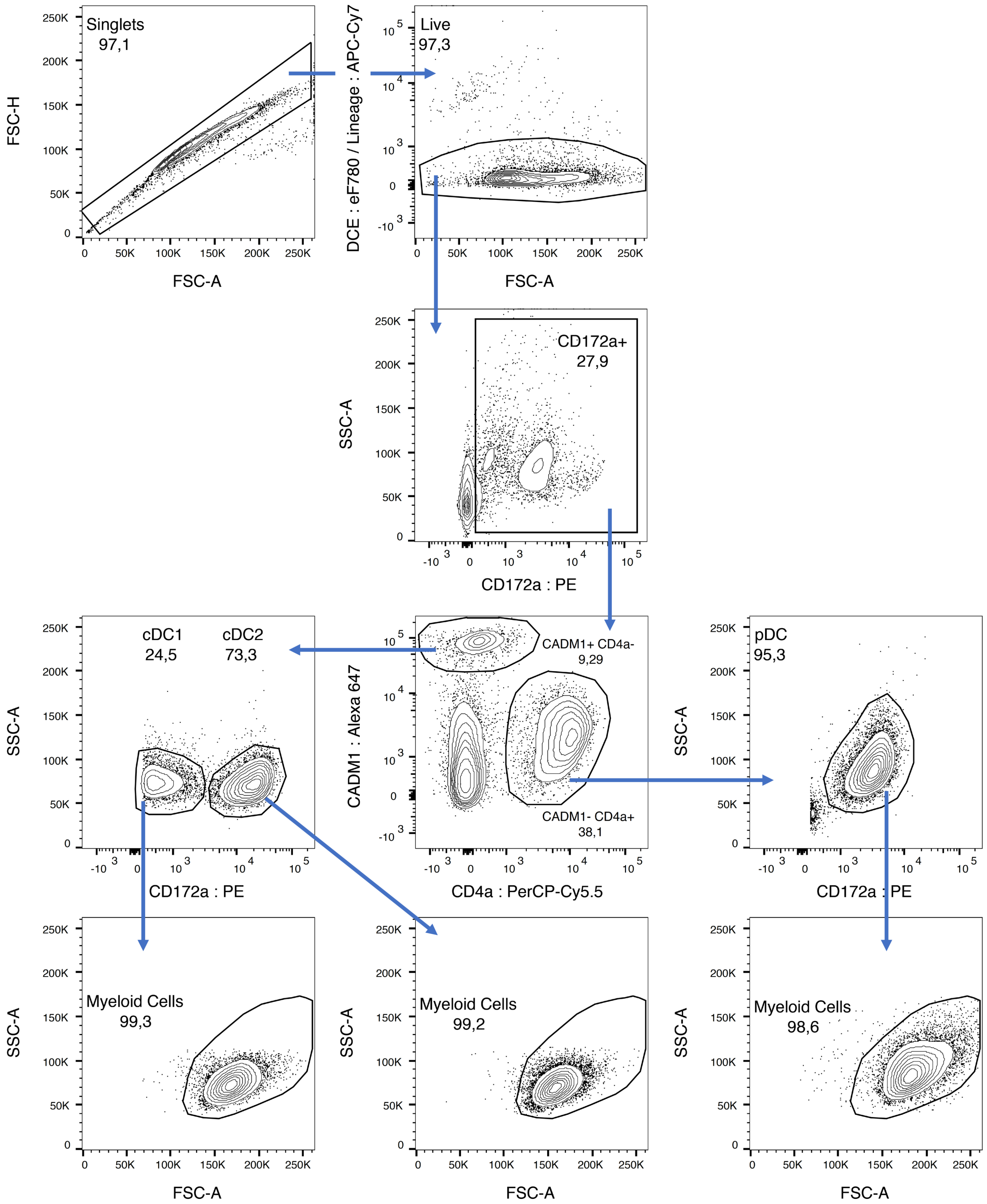

Supplementary Figure 5: Modified version of the gating strategy displayed in Figure 1A, excluding pre-gating on myeloid cells based on size and granularity (FSC-A and SSC-A). The live cell gate has been expanded here to include small (FSC-A low) cells which are excluded by the myeloid cell gate in Figure 1A. All other gates are identical. After identification of DC subsets based on cell surface marker expression, each subset is displayed here within the same myeloid cell gate used in Figure 1A. This demonstrates that almost all DC (cDC1: 99.3%; cDC2: 99.2%; pDC: 98.6%) fall within the myeloid cell gate, and the inclusion of this gate during sorting did not exclude significant numbers of DC.
